# Major Sources of Organic Matter in a Complex Coral Reef Lagoon: Identification from Isotopic Signatures (δ^13^C and δ^15^N)

**DOI:** 10.1371/journal.pone.0131555

**Published:** 2015-07-02

**Authors:** Marine J. Briand, Xavier Bonnet, Claire Goiran, Gaël Guillou, Yves Letourneur

**Affiliations:** 1 Université de la Nouvelle-Calédonie, Laboratoire LIVE and LABEX « Corail », BP R4, 98851 Nouméa cedex, New Caledonia; 2 Centre d’Etudes Biologiques de Chizé, UMR 7372 CNRS-ULR, 79360 Villiers-en-bois, France; 3 Université de La Rochelle, Département Littoral Environnement et Sociétés, UMR CNRS 7266 LIENSs, Bât. Marie Curie, Rue Olympe de Gouges, 17042 La Rochelle cedex 1, France; Biodiversity Research Center, Academia Sinica, TAIWAN

## Abstract

A wide investigation was conducted into the main organic matter (OM) sources supporting coral reef trophic networks in the lagoon of New Caledonia. Sampling included different reef locations (fringing, intermediate and barrier reef), different associated ecosystems (mangroves and seagrass beds) and rivers. In total, 30 taxa of macrophytes, plus pools of particulate and sedimentary OM (POM and SOM) were sampled. Isotopic signatures (C and N) of each OM sources was characterized and the composition of OM pools assessed. In addition, spatial and seasonal variations of reef OM sources were examined. Mangroves isotopic signatures were the most C-depleted (-30.17 ± 0.41 ‰) and seagrass signatures were the most C-enriched (-4.36 ± 0.72 ‰). *Trichodesmium* spp. had the most N-depleted signatures (-0.14 ± 0.03 ‰) whereas mangroves had the most N-enriched signatures (6.47 ± 0.41 ‰). The composition of POM and SOM varied along a coast-to-barrier reef gradient. River POM and marine POM contributed equally to coastal POM, whereas marine POM represented 90% of the POM on barrier reefs, compared to 10% river POM. The relative importance of river POM, marine POM and mangroves to the SOM pool decreased from fringing to barrier reefs. Conversely, the relative importance of seagrass, *Trichodesmium* spp. and macroalgae increased along this gradient. Overall, spatial fluctuations in POM and SOM were much greater than in primary producers. Seasonal fluctuations were low for all OM sources. Our results demonstrated that a large variety of OM sources sustain coral reefs, varying in their origin, composition and role and suggest that δ^13^C was a more useful fingerprint than δ^15^N in this endeavour. This study also suggested substantial OM exchanges and trophic connections between coral reefs and surrounding ecosystems. Finally, the importance of accounting for environmental characteristics at small temporal and spatial scales before drawing general patterns is highlighted.

## Introduction

Marine primary producers are the basis of the functioning of coastal and pelagic ecosystems [1]. At the ocean scale phytoplanktonic production is largely responsible for organic carbon input into the food webs [2], whereas at the scale of a coastal ecosystem macrophyte algae (*sensu lato)* play a much greater role in the organic carbon input [3]. Tropical coral reefs are complex, highly diversified ecosystems with numerous potential sources of organic matter (OM), and the major primary producers on reefs are benthic macroalgae, including turf algae. Primary producers of reef-associated ecosystems such as mangroves and seagrass beds also provide a significant amount of organic carbon and other elements such as nitrogen. For instance, 1–100% of leaves may be exported from seagrass beds as organic matter, with an average of about 25% [4]. Similarly, approximately 30–50% of leaves may be exported from mangroves [5].

The major organic sources in an ecosystem are generally mixed together into global pools of OM [6]. These highly heterogeneous pools are distributed in the water and in the sediments and contain both living and dead organic materials from various origins; this is especially true for coastal zones. The particulate organic matter (POM) in water is a mixture of phytoplankton, bacteria, invertebrates and fish fecal pellets, and detrital particles [7]. Mainly detrital, the sedimentary organic matter (SOM) contains all of the above components plus the micro-phytobenthos and the meiofauna [8]. In some coastal zones SOM may also contain substantial continental inputs from river sediment [9]. These complex pools contain organic material produced by very different organisms using various photosynthetic processes. Teasing apart their respective contributions to ecosystem functioning remains difficult.

A solution to this problem may be provided by the use of isotopic signatures, as they allow discrimination of the various OM sources [10]. For example, terrestrial or marine OM origins can be identified by stable isotopes (specifically δ^13^C and δ^15^N), from which the relative OM contributions to SOM and/or POM pools can also be assessed [11]. This approach has been widely applied in temperate coastal zones [12–14], but has rarely been used in coral reefs [9]. Consequently, information about the functioning of highly diversified ecosystems such as coral reefs remains fragmentary.

Although partitioning the different OM sources among primary consumers largely underpins the transit of OM through food webs, studies that have investigated spatial and temporal fluctuations of OM sources using isotopic signatures have mostly focused on only one or a few primary producers [15, 16]. However, it is well known that numerous species feed on macroalgae, turf algae and seagrass leaves [17, 18]. These plants represent the main source of energy for shallow coastal ecosystems and play an important role in benthic nutrient recycling. POM and SOM also influence ecosystem functioning as they transit through trophic networks from the moment they are consumed by primary producers [19]. To properly assess OM origin and flow across coral reefs trophic networks it is necessary to simultaneously study the respective isotopic signatures of POM, SOM, macroalgae and seagrass.

Our present study aimed at characterizing the isotopic signatures (δ^13^C and δ^15^N) of several potential OM sources in coral reef ecosystems. The wide, diversified and complex coral reef lagoon of New Caledonia, south-west Pacific, was used as a study case. The first objective was to assess the diversity of potential OM sources and their relative contributions to the pools of OM (POM and SOM). The second objective was to examine the spatio-temporal variations of isotopes δ^13^C and δ^15^N for the most common reef OM sources in selected lagoonal locations along two coast-to-ocean gradients and over two seasons.

## Material and Methods

### Study sites and data sampling / collection

**Fig 1:**
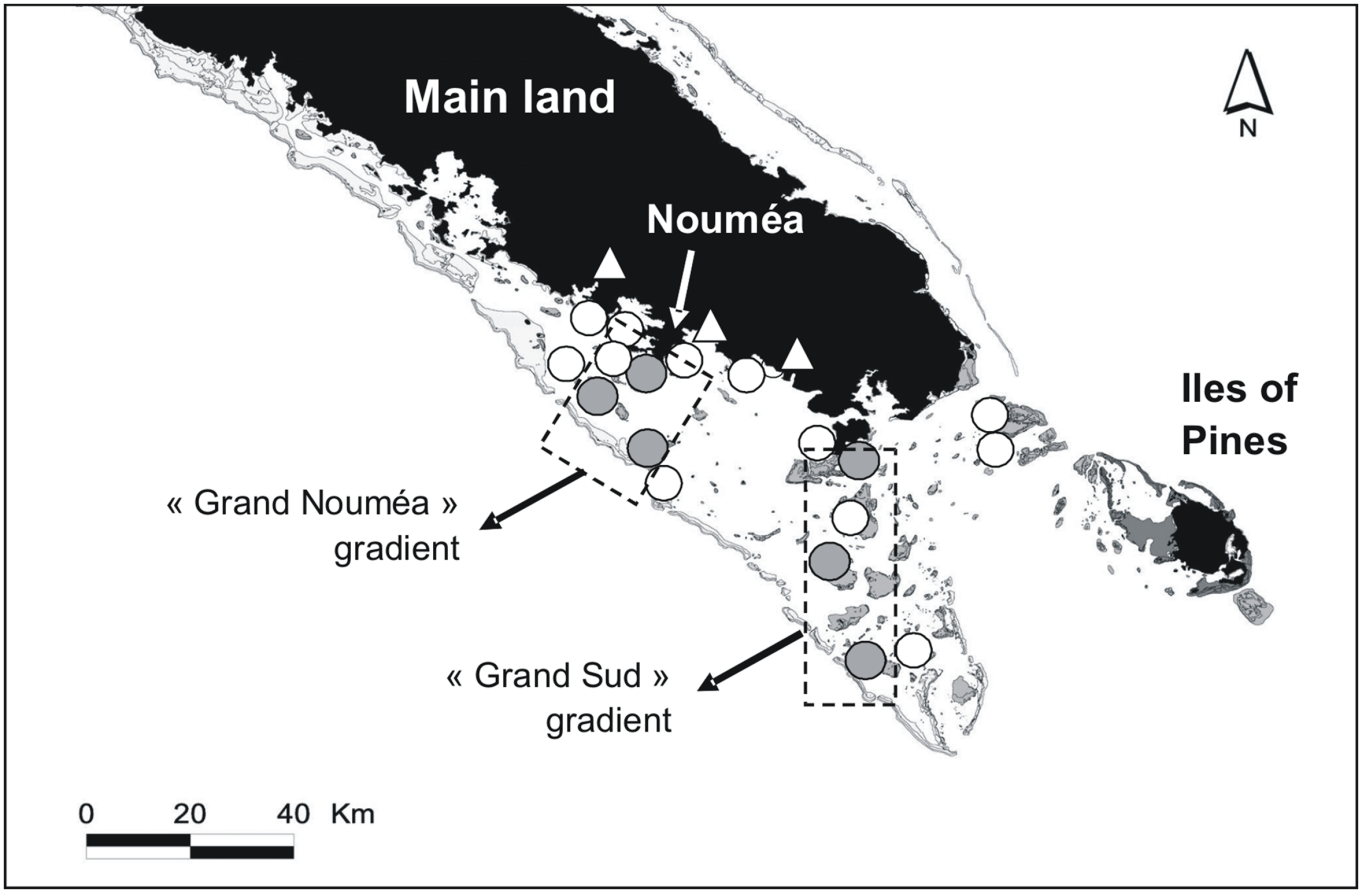
Study sites locations in the southwest lagoon of New Caledonia, SW Pacific Ocean. White marks: OM sources sampled for the first objective in rivers (triangles) and lagoon sites (circles). Grey circles: OM sources sampled in lagoon sites along the two coast-to-ocean gradients for the second objective.

The study site is situated in the southern part of the New Caledonian lagoon (SW Pacific Ocean) (Fig 1). Habitats encompassing various marine landscapes were sampled: mangroves, seagrass beds, coastal soft-bottoms (i.e. without any rocky or coral structure) and coral reefs. The latter were represented by fringing reefs (i.e. close to the shoreline), intermediate reefs (i.e. around islets located in the middle of the lagoon) and barrier reefs separating the lagoon from the open ocean.

In March and April 2010, OM sources from each marine habitat were sampled at various locations in shallow waters, from 0.5 to 5 m deep, and with a mean water temperature of 26°C (Fig 1). A total of 605 samples of OM sources were collected; 27 different macroalgae (including turf algae; see below) and seagrass species, two mangrove species, plus POM and SOM from the superficial subsurface layer of water (< 10 cm) and sediment (< 3 cm) respectively. Subsurface freshwater samples from there small rivers were also collected, approximately 5–10 km from the river mouth and at low tide to avoid sampling any marine water. Marine water POM was collected near passes whereas coral reef POM was collected on fringing, intermediate and barrier reefs. No specific permissions were required for these locations for such kind of samples that did not involve endangered or protected species.

In summer 2011, hurricane Vania (14^th^ January) and the moderate depression Zellia (17^th^ January) hit New Caledonia. This generated strong rainfall, causing unusually large ground erosion and large amounts of freshwater POM flowed into the lagoon. Freshwater samples were collected just after the major flooding event in order to assess the possible influence of freshwater POM on isotopic (C-N) signatures. In March 2011, a large *Trichodesmium* spp. bloom occurring in lagoon waters was also sampled. These algae are known to play important role in nitrogen fixation [20]. Flood and bloomevents offered excellent opportunities to examine the impact of unusual events on the isotopic (C-N) signatures of the studied ecosystem (e.g. subsurface seawaters δ^15^N signature).

In February-April (summer) and August-September (winter) 2011 some common OM sources were sampled again along two coast-to-ocean gradients in the lagoon (i.e. fringing, intermediate and barrier reefs on the “Grand Nouméa” and “Grand Sud” gradients, Fig 1); Nineteen different macroalgae and seagrass species were collected, plus POM and SOM. Turf algae were accounted for as part of the macrophytes sources, as they are one of the major sources of OM on coral reefs [9, 21]. Turf algae will be hereafter referred as algal “turfs”. Algal turfs correspond here to the complex algal species assemblage dominated by Ceramials and usually found in pomacentrid’ territories [22, 23]. The most dominant species of macroalgae and seagrass were sampled when they were accessible. Dominance is here defined in terms of abundance or percentage of substrate cover. Subsurface seawater was collected at each site at mid-tides in order to capture the averaged signal of POM and to avoid a biased signal (at high tides the marine influence is high, whereas at low tides the terrigeneous signal is potentially stronger).

### Stable isotope preparation

All subsurface fresh- and seawater samples were filtered on pre-weighted Whatman GF/F filters (porosity 0.7 μm) pre-combusted for 4 h at 450°C. The 63–200 μm-sized fraction was considered to be the best proxy for analyzing the main phytoplankton components of the community [24, 25]. The present study focused on obtaining broad isotopic signatures of fresh- and seawater POM, rather than analyzing the various fractions of phytoplankton. For instance, the smallest components of phytoplankton, namely pico- and nanoplankton, were not taken into account in this study. However, the largest particles and detritus were removed in order to avoid bias in isotopic values.

Seawater and freshwater POM samples collected on GF/F filters were freeze-dried and cut into small pieces. Vegetal and sediment samples were freeze-dried and ground into a fine powder (< 6 μm) using a mortar and pestle. Calcareous macrophytes, marine and coral reef POM and SOM were divided into two sub-samples each. One sub-sample was allocated to the carbon isotope analysis; it was acidified with 1% HCl solution to remove carbonates, rinsed with distilled water and oven-dried at 40°C for 24 h. This protocol is in agreement with the carbonates’ higher δ^13^C in comparison to organic carbon [26]. The other sample was allocated to the nitrogen isotope analysis; it was not acidified to prevent undesirable enrichment in ^15^N [27]. Samples from non-calcareous macrophytes and from freshwater POM were analysed without any pre-treatment. Considering the thickness of sediments collected (superficial subsurface layer), we assumed that isotopic values of SOM samples were not biased by any partial remineralisation through bacterial activity.

The ^13^C:^12^C and ^15^N:^14^N ratios were analyzed by continuous-flow isotope-ratio mass spectrometry. The spectrometer (Delta V Plus stable-isotope analyzer coupled with a Falsh EA 2000 analyzer; Thermo Scientific, Bremen, Germany) was operated in dual isotope mode. The analytical precision was <0.15% for both N and C, estimated from standards analyzed along with the samples. Internal standards were 1 mg leucine calibrated against ‘Europa flour’ and IAEA standards N1 and N2. Isotope ratios were expressed as parts per 1000 (‰) differences from a standard reference material:

δX = [(R_sample_ x R^-1^_standard_) − 1] x 10^3^; where X is ^13^C or ^15^N, R is the corresponding ratio (^13^C:^12^C or ^15^N:^14^N) and δ is the measure of heavy to light isotope in the sample. The international standard references are Vienna Pee Dee Belemnite (vPDB) for carbon and atmospheric N_2_ for nitrogen.

### Data analyses

#### Comparison tests

The Levene test was run on the variances of OM sources (i.e. POM, SOM and macrophytes) to assess their homogeneity prior their analysis, and consequently *t*-tests were performed to compare among means [28]. In the event of heterogeneous variances, non-parametric Kruskall–Wallis tests were run for the analyses. When a source had been sampled during only one season, only spatial analyses were conducted and ANOVAs tests or non-parametric Kruskall-Wallis tests were used for this purpose.

#### Assessments of sources of potential contributions to POM and SOM pools

Different models can be used to evaluate the contribution of various sources to an organic pool, including Bayesian mixing-models [29]. Irrespective of the method used, a pool isotopic signature is considered as the mean of the signatures of the various constitutive or incorporated sources [30]. For instance, in a pool made of three potential sources, where each source has specific δ^13^C and δ^15^N signature, the resulting signature of the pool is expressed as follow [31]:

$$\delta^{13}C_{pool}=f_{1}\delta^{13}C_{1}+f_{2}\delta^{13}C_{2}+f_{3}\delta^{13}C_{3}$$

$$\delta^{15}N_{pool}=f_{1}\delta^{15}N_{1}+f_{2}\delta^{15}N_{2}+f_{3}\delta^{15}N_{3}$$

$$f_{1}+f_{2}+f_{3}=1$$

where δ^13^C*i* and δ^15^N*i* are the isotopic signatures for sources 1 to 3 and *f* is the relative proportion of the contribution of a source to the pool.

The relative contributions of various OM sources to POM and SOM pools in different habitats were here assessed with Bayesian mixing-models (R software and SIAR package [32]). Firstly, the relative contribution of the river POM, marine POM, and *Trichodesmium* spp. blooms to the particulate OM from lagoon waters at fringing, intermediate and barrier reef sites was examined. Secondly, the relative contribution of mangrove leaves, seagrass, macroalgae (algal turf, calcareous Chlorophytae, Phaeophycae and Rhodophytae), *Trichodesmium* spp., river POM and marine POM on the sedimentary OM from all habitats (mangroves, coastal soft-bottoms, fringing reefs, intermediate reefs and barrier reefs) was determinded.

These models calculated the most feasible solutions to explain isotopic ratios measured for POM or SOM and they also allowed integrating all uncertainties related to the OM sources. A major issue with the use of mixing models lies in the choice of trophic enrichment factors (TEFs), which strongly influence the models outputs [33]. TEF was set to null as no consumption process was involved and only the mix of several potential sources of OM was considered.

## Results

### Diversity of organic matter sources

**Fig 2:**
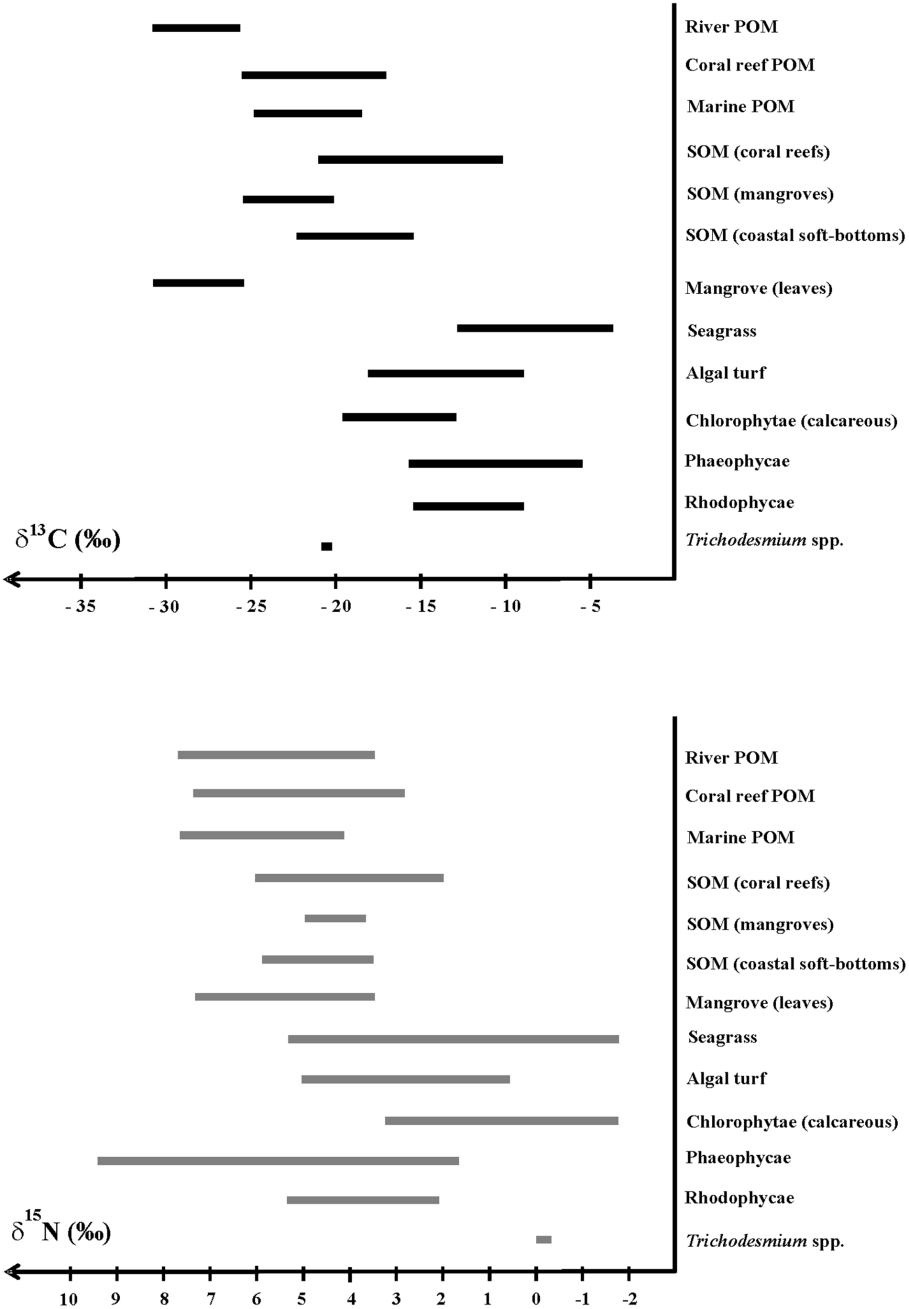
Ranges of isotopic signature values (top: δ^13^C; bottom: δ^15^N) for the major OM sources in the SW lagoon of New Caledonia. All the data sampled were pooled into one single data set.

River POM was the most ^13^C-depleted pool (δ^13^C from -30.78 to -26.67 ‰, Fig 2). Pre- and post-hurricane δ^13^C and δ^15^N signatures were not significantly different (p> 0.75 in both cases) and therefore these data were pooled. Coral reef SOM (fringing, intermediate and barrier reefs) was the most ^13^C-enriched pool (δ^13^C from -22.16 to -10.28 ‰). Marine and coral reef POM, mangrove SOM and soft-bottom SOM pools varied at intermediate levels between these extremes (Fig 2). River POM, marine and coral reef POM and coral reef SOM showed relatively similar ranges of δ^15^N signatures, but with minimal and maximal isotopic signatures being lower for the latter (Fig 2). Mangrove SOM and soft-bottom SOM δ^15^N signatures overlapped in value, with the range of the former being narrower.

**Fig 3:**
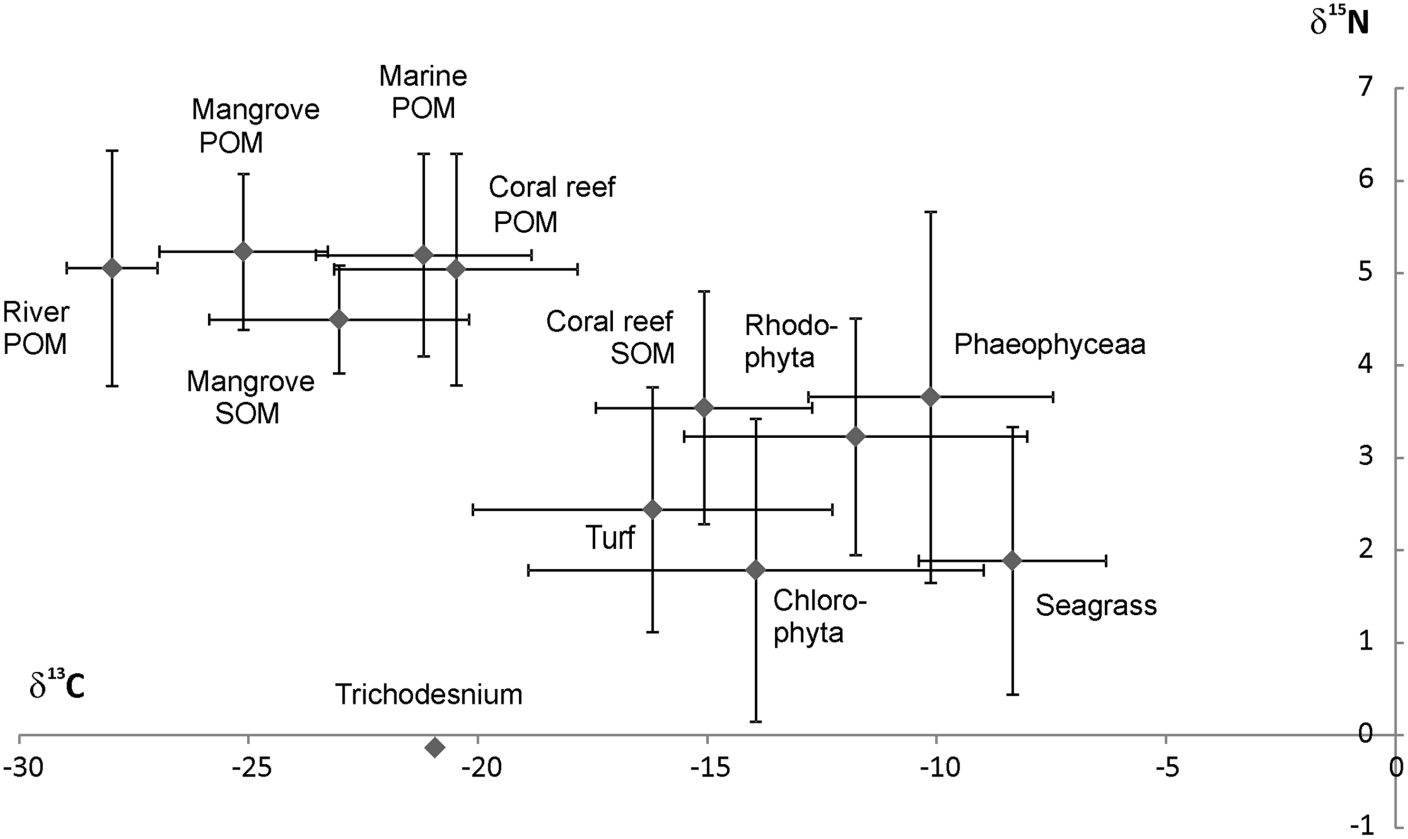
δ^13^C *versus* δ^15^N scatterplot of major OM sources in the SW lagoon of New Caledonia.

Among primary producers, mangrove was the most ^13^C-depleted (from -30.95 to -25.91 ‰) and seagrass was the most ^13^C-enriched (from -13.21 to -3.50 ‰). Algal turfs (from -17.87 to -7.54 ‰), Chlorophytea (from -20.79 to -4.73 ‰), Phaeophycea (from -16.47 to -4.26 ‰), and Rhodophytea (from -13.21 to -5.83 ‰) displayed intermediate values (Fig 2). *Trichodesmium* spp. signatures were in narrow ranges for both δ^13^C (from -21.03 to -20.78 ‰) and δ^15^N (from -0.36 to 0.02 ‰). Overall, the primary producers’ δ^15^N signatures typically overlapped. This overlap was due to strong inter-species variations in each primary producer category (Table 1, Fig 3). For instance, within the seagrass group, *Synrigodium iseotifolium* displayed a significantly higher δ^13^C value and *Cymodocea rotondata* presented a significantly lower δ^15^N value compared to the other seagrass species (Table 1). Similarly, in the Chlorophytae group, *Halimeda cylindracea* δ^15^N value was significantly lower than the *H*. *discoïdea* signature (Table 1).

**Table 1:** Mean δ^13^C and δ^15^N isotopic ratios (± sd) of primary producers sampled in the SW lagoon of New Caledonia (data from 2010 and 2011 pooled).

Category	Species	*N*	δ^13^C (‰)	δ^15^N (‰)
Algal turf		*54*	-16.19 ± 2.92	2.44 ± 1.33
Chlorophytae	*Halimeda borneensis*	*14*	-14.87 ± 3.41	1.29 ± 1.52
	*H*. *cylindracea*	*39*	-11.80 ± 3.22	0.33 ± 1.75
	*H*. *discoïdea*	*15*	-14.52 ± 3.02	3.62 ± 1.36
	*H*. *heteromorpha*	*3*	-16.85 ± 0.63	1.51 ± 0.40
	*H*. *macroloba*	*12*	-13.14 ± 0.98	0.89 ± 0.79
	*H*. *micronesica*	*6*	-11.35 ± 2.97	2.22 ± 0.48
	*H*. *opuntia*	*50*	-15.89 ± 4.53	2.35 ± 0.93
Phaeophycae	*Cystoseira* sp.	*6*	-12.16 ± 0.97	3.09 ± 1.16
	*Padina australis*	*20*	-8.07 ± 1.33	3.47 ± 1.82
	*Sargassum cristaefolium*	*3*	-13.93 ± 0.92	2.36 ± 0.24
	*S*. *spinuligerum*	*20*	-12.84 ± 2.11	5.15 ± 1.67
	*Sargassum* sp.	*11*	-14.60 ± 1.19	3.91 ± 0.20
	*Turbinaria conoïdes*	*14*	-9.35 ± 1.39	2.88 ± 0.90
	*T*. *ornata*	*15*	-9.69 ± 1.20	3.43 ± 1.52
	*Turbinaria* sp.	*3*	-7.44 ± 0.84	2.86 ± 0.31
Rhodophytae	*Acanthophora spicifera*	*3*	-11.71 ± 0.62	2.09 ± 0.22
	*Digenea simplex*	*3*	-14.76 ± 1.13	4.54 ± 0.14
	*Hormophysa cuneiformis*	*6*	-12.90 ± 0.49	3.15 ± 0.59
	*Laurencia* sp.	*15*	-13.38 ± 2.52	3.29 ± 1.57
	*Liagora* sp.1	*6*	-3.16 ± 1.20	2.75 ± 0.30
	*Liagora* sp.2	*6*	-6.29 ± 0.71	2.68 ± 0.24
	*Lobophora variegata*	*3*	-12.56 ± 1.04	3.92 ± 0.07
Seagrass	*Cymodocea rotundata*	*6*	-7.24 ± 0.64	0.64 ± 1.54
	*C*. *serrulata*	*18*	-9.39 ± 1.60	2.97 ± 1.22
	*Halodule uninervis*	*27*	-8.55 ± 1.24	1.66 ± 1.33
	*Halophila ovalis*	*6*	-7.78 ± 1.95	1.20 ± 0.23
	*Synrogodium iseotifolium*	*6*	-4.36 ± 0.72	1.57 ± 1.16
Mangrove tree	*Avicenia marina*	*3*	-27.26 ± 0.30	6.47 ± 0.41
	*Rhizophora stylosa*	*6*	-30.17 ± 0.41	3.72 ± 0.25

### Relative contributions of organic matter sources to POM pool

**Fig 4:**
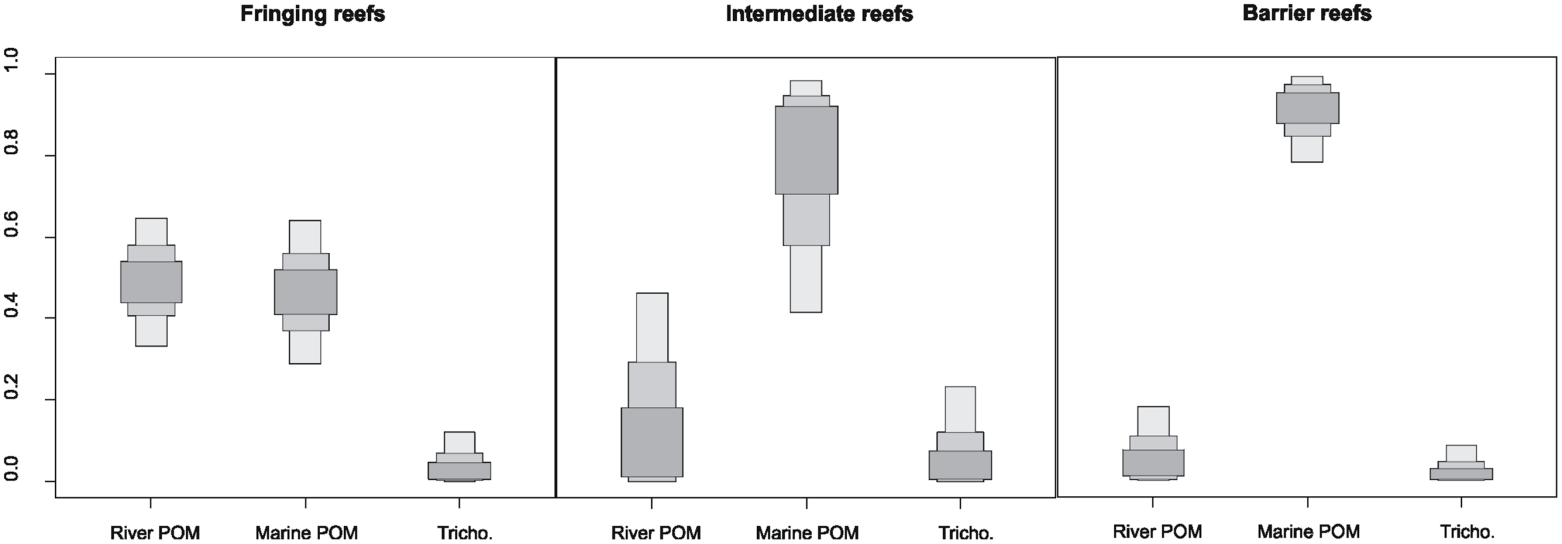
Relative importance of river POM, marine POM and *Trichodesmium* spp. (Tricho.) in the isotopic composition of coral reef POM on fringing, intermediate, and barrier reefs. Shaded boxes represent, from dark to light grey, 50%, 75%, and 95% Bayesian credibility intervals.

Following the coast to barrier reef gradient the influence of river POM clearly decreased whereas the influence of marine POM increased (Fig 4). Both the Grand Nouméa gradient and Grand Sud gradient displayed a similar pattern (S1 Fig). At coastal sites, the POM pool was 50% composed of river POM and 50% of marine POM; the Grand Nouméa gradient showed a slightly higher contribution of river POM than the Grand Sud gradient (55–60% compared to 40%). At barrier reef sites, the POM pool was dominated by marine POM (90% compared to 10% for river POM). The relative contribution of *Trichodesmium* spp. to POM was typically less than 10% (Fig 4), although on some of the Grand Sud gradient reefs it reached approximately 15% (S1 Fig).

Some components of POM are missing in our approach, such as nano- and picophytoplankton because they cannot be collected on GF/F filters. Consequently, some potential sources of POM are missing from this study.

### Relative contributions of organic matter sources to SOM pool

**Fig 5:**
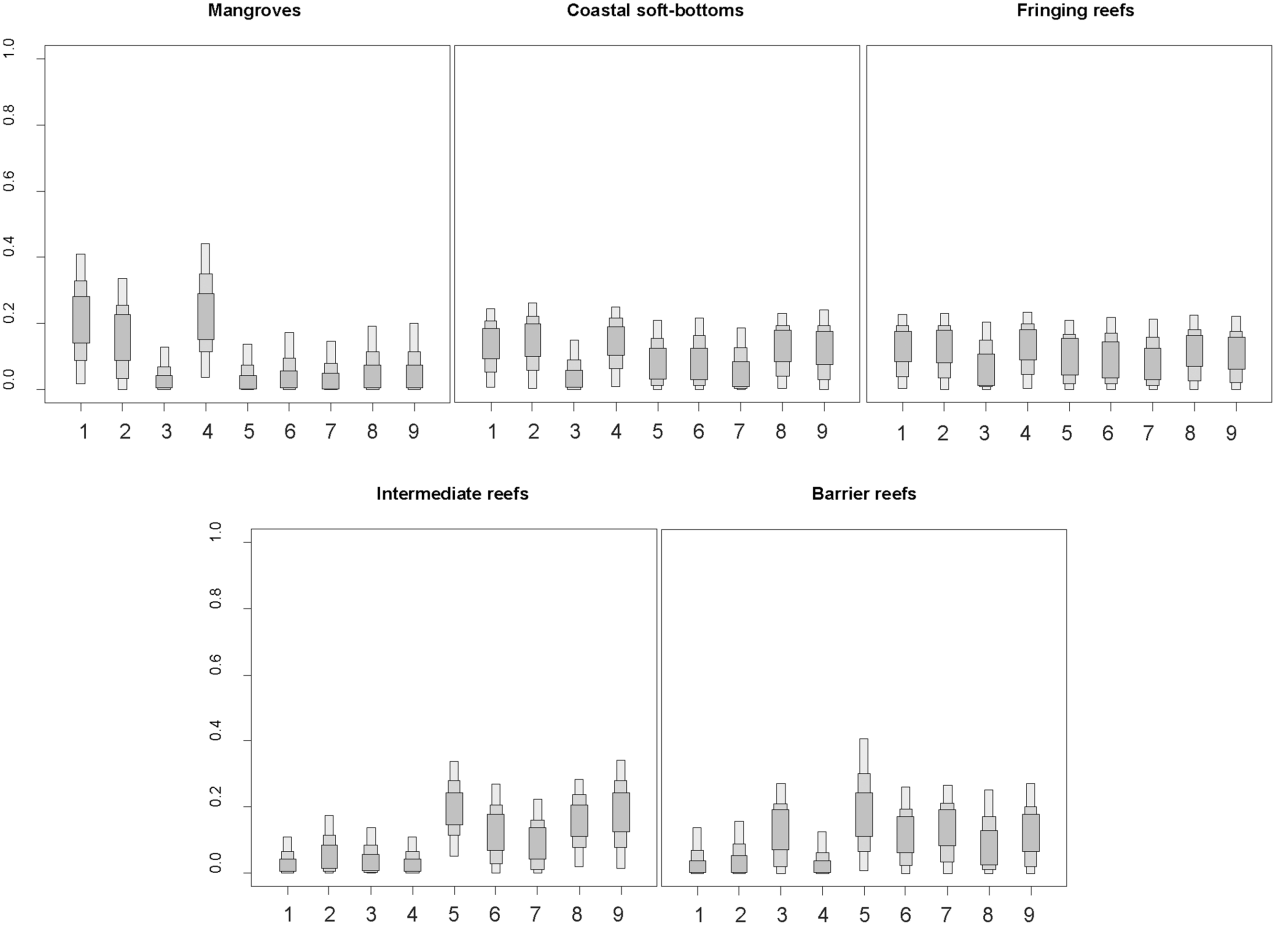
Relative importance of various OM sources in the isotopic composition of SOM in mangroves, coastal soft-bottoms, fringing, intermediate, and barrier reefs. Sources codes: (1) river POM, (2) marine POM, (3) *Trichodesmium* spp., (4) mangroves leaves, (5) seagrass, (6) algal turf, (7) Chlorophytae (calcareous), (8) Phaeophycae, and (9) Rhodophytae. Shaded boxes represent 50%, 75% and 95% Bayesian credibility intervals from dark to light grey.

All OM sources contributed to the SOM pool, with variations among habitats and along the coast-to-ocean gradient (Fig 5). River POM, marine POM and mangroves’ relative contributions to SOM pool decreased from coast to barrier reef. Conversely, the relative importance of seagrass, *Trichodesmium* spp., and to a lesser extent macroalgae, progressively increased along this gradient (Fig 5). Approximately 60% of mangrove SOM derived from mangrove leaves (approximately 25%), river POM (approximately 20%) and marine POM (approximately 15%); the other sources contributed to less than 10%. Fringing reefs and coastal soft-bottom SOM isotopic signatures were relatively similar. Their compositions were diverse, with each source contributing 5% to 15% (Fig 5). Intermediate and barrier reefs SOM mainly derived from algal turf, seagrass and macroalgae, with proportions of each ranging from approximately 10% to over 20%. *Trichodesmium* spp. was also important on barrier reefs, where it contributed to approximately 15% of the SOM isotopic signature (Fig 5).

### Variations of δ^13^C and δ^15^N isotopic signatures among the main habitats

Mangroves and seagrass OM displayed significantly different δ^13^C and δ^15^N values (p < 0.001; Tables 1 and 2). On coral reefs, algal turf and macroalgae primary producers’ δ^13^C isotopic signatures were statistically similar (p > 0.05). However, they were significantly different to seagrass and mangroves signatures (p < 0.05; Table 2). δ^15^N signatures alone were not sufficient to discriminate the various OM sources (Table 2). δ^13^C and δ^15^N *Trichodesmium* spp. signatures were significantly different from all the other primary producers (p < 0.005; Table 2).

**Table 2:** Mean isotopic signatures (± sd) in carbon (δ^13^C) and nitrogen (δ^15^N) for the major sources of organic matter in different habitats of the SW lagoon of New Caledonia.

Source	Habitat	δ^13^C (‰)	δ^15^N (‰)	*N*
POM	River	-27.98 ± 0.99	5.05 ± 1.27	*15*
	Mangrove	-25.11 ± 1.83	5.23 ± 0.58	*6*
	Coastal soft bottom	-22.57 ± 1.46	5.22 ± 0.53	*9*
	Marine	-21.10 ± 2.45	5.34 ± 0.98	*6*
	Coral reefs	-20.58 ± 2.65	5.04 ± 1.26	*60*
SOM	Mangrove	-23.02 ± 2.83	4.50 ± 0.58	*6*
	Coastal soft bottom	-17.88 ± 3.61	3.87 ± 0.52	*9*
	Coral reefs	-15.70 ± 2.35	3.54 ± 1.26	*60*
Mangrove’ leaves	Mangrove	-28.22 ± 1.68	5.36 ± 1.31	*9*
Seagrass	Mangrove	-11.42 ± 0.92	2.49 ± 1.94	*6*
Seagrass	Coral reefs	-8.35 ± 2.04	1.88 ± 1.45	*63*
Algal turf	Coral reefs	-16.19 ± 3.92	2.44 ± 1.33	*54*
Chlorophytae	Coral reefs	-13.94 ± 3.69	1.78 ± 1.84	*148*
Phaephocycea	Coral reefs	-10.13 ± 2.67	3.66 ± 2.01	*92*
Rhodophytae	Coral reefs	-11.99 ± 2.69	3.28 ± 1.31	*43*
Rhodophytae	Mangrove	-14.64 ± 1.91	3.85 ± 0.86	*6*
*Trichodesmium* spp.	Lagoon waters	-20.94 ± 0.02	-0.14 ± 0.03	*6*

POM = particulate organic matter, SOM = sedimentary organic matter (data from 2010 and 2011 pooled).

POM and SOM pools displayed significantly different δ^13^C signatures among habitats. River POM was significantly ^13^C-depleted compared to coral reef POM and to coral reef SOM (p < 0.001; Table 2). δ^15^N signatures were similar between coral reef POM and river POM, but both differed significantly from coral reef SOM (p < 0.001). POM and SOM pools displayed lower δ^13^C values and higher δ^15^N values than most primary producers (Table 2). River POM displayed similar C and N isotopic signatures to those of mangrove leaves (p > 0.05). The coral reef POM δ^13^C signature was significantly different from those of all primary producers (p < 0.05), except *Trichodesmium* spp. Coral reef POM δ^15^N value did not differ statistically from those of mangrove leaves and Chlorophytae (p > 0.05). Coral reef SOM δ^13^C and δ^15^N signatures significantly differed from those of mangrove leaves, mangrove SOM, and seagrass (p < 0.05 for all cases).

### Spatial patterns of δ^13^C and δ^15^N isotopic signatures

Isotopic signatures of some OM sources significantly varied along the coast-to-ocean gradient (Table 3). POM and SOM pools fluctuate spatially more than primary producers; this was mainly for δ^13^C signatures, and the δ^15^N algal turf signature. POM δ^13^C signatures were significantly lower on barrier reefs than on fringing and intermediate reefs, whereas POM δ^15^N signatures were significantly higher on barrier reefs (Tables 3 and 4). These trends were found on Grand Nouméa and Grand Sud, despite some specific differences (S1 Table). SOM δ^13^C signatures were, overall, significantly lower on fringing reefs and significantly higher on intermediate reefs, whereas SOM δ^15^N signatures were significantly higher on barrier reefs (Tables 3 and 4). However, this general trend on both coast-to-ocean gradients displayed noticeable differences for intermediate (δ^13^C) or barrier reefs (δ^15^N) (S2 Table).

**Table 3:** Mean isotopic signatures (± sd) in carbon (δ^13^C) and nitrogen (δ^15^N) for the different sources of organic matter sampled along coast-to-ocean gradients, both zones and seasons pooled, in the SW lagoon of New Caledonia in 2011. Only sources present on at least two reef types were shown.

Category	Species	Site	δ^13^C (‰)	δ^15^N (‰)	*N*
POM		FR	-18.90 ± 1.70	4.85 ± 0.71	*14*
		IR	-18.98 ± 1.33	3.79 ± 0.59	*12*
		BR	-20.82 ± 1.91	5.62 ± 0.75	*12*
SOM		FR	-15.73 ± 0.72	3.59 ± 0.56	*13*
		IR	-13.33 ± 0.85	2.30 ± 0.25	*14*
		BR	-14.77 ± 3.35	4.45 ± 2.17	*14*
Algal turf		FR	-18.41 ± 2.15	2.67 ± 0.27	*6*
		IR	-18.49 ± 2.33	1.94 ± 0.39	*12*
		BR	-19.48 ± 2.06	3.05 ± 0.99	*12*
Chlorophytae	*Halimeda borneensis*	FR	-12.99 ± 0.16	1.93 ± 0.13	*3*
		IR	-13.33 ± 1.52	0.16 ± 1.13	*9*
		BR	-18.76 ± 0.19	3.34 ± 0.15	*3*
	*H*. *cylindracea*	FR	-16.05 ± 2.13	0.91 ± 0.17	*6*
		IR	-12.14 ± 1.31	0.52 ± 1.59	*9*
		BR	-14.23 ± 0.50	0.82 ± 3.52	*6*
	*H*. *discoïdea*	IR	-13.95 ± 2.68	2.33 ± 1.29	*10*
		BR	-15.37 ± 1.56	5.77 ± 1.21	*6*
	*H*. *opuntia*	FR	-18.75 ± 1.00	2.18 ± 0.30	*9*
		IR	-18.05 ± 2.63	1.72 ± 0.63	*12*
		BR	-19.38 ± 0.90	3.07 ± 1.56	*9*
Phaeophycae	*Padina australis*	FR	-7.31 ± 0.94	2.08 ± 0.15	*6*
		IR	-7.61 ± 0.77	2.11 ± 0.54	*8*
	*Sargassum spinuligerum*	FR	-14.00 ± 2.00	3.17 ± 0.13	*3*
		IR	-12.46 ± 2.25	5.89 ± 1.29	*9*
	*Turbinaria conoïdes*	IR	-8.71 ± 1.23	2.84 ± 1.10	*8*
		BR	-10.91 ± 0.80	1.78 ± 0.21	*3*
	*T*. *ornata*	IR	-10.56 ± 1.96	2.25 ± 0.52	*6*
		BR	-9.47 ± 0.93	3.73 ± 1.55	*12*
Seagrass	*Cymodocea serrulata*	FR	-9.51 ± 0.35	2.64 ± 0.86	*6*
		IR	-8.32 ± 0.14	3.04 ± 0.56	*6*
	*Halodule uninervis*	IR	-8.01 ± 0.67	2.58 ± 0.91	*6*
		BR	-8.79 ± 0.96	1.06 ± 0.50	*6*

POM = particulate organic matter, SOM = sedimentary organic matter, FR = fringing reef, IR = intermediate reef and BR = barrier reef.

**Table 4:** Summary of the significant spatial variability of isotopic signatures (δ^13^C and δ^15^N) of the sources of organic matter in the SW lagoon of New Caledonia in 2011.

Sources	Factors	δ^13^C	δ^15^N
POM	*Site*	***	***
	*zone x site*	***	*
	*season*	***	ns
	*zone x site x season*	***	*
SOM	*Site*	***	***
	*zone x site*	***	***
	*season*	***	ns
	*site x season*	***	*
Algal turf	*site*	ns	***
	*zone x site*	ns	***
	*season*	**	***
	*site x season*	***	***
	*zone x site x season*	*	***
*Halimeda borneensis*	*site*	***	***
	*season*	ns	*
*H*. *cylindracea*	*site*	***	ns
	*zone x site*	**	***
	*season*	**	**
	*site x season*	***	ns
*H*. *opuntia*	*site*	*	***
	*zone x site*	ns	***
	*season*	*	ns
	*site x season*	***	**
*H*. *discoïdea*	*site*	***	***
	*season*	**	***
*Sargassum spinuligerum*	*site*	ns	*
*Turbinaria conoides*	*site*	ns	**
*T*. *ornata*	*site*	*	ns
*Cymodocea serrulata*	*site*	**	ns
*Halodule uninervis*	*site*	ns	***
	*season*	*	**

Analyses were run with three-way ANOVAs or Kruskal-Wallis tests: zone (i.e. Grand Nouméa *versus* Grand Sud) x site (fringing *versus* intermediate *versus* barrier reef) x season (summer *versus* winter).

ns = p> 0.05

* p< 0.05

** p< 0.01

*** p< 0.001.

POM = particulate organic matter, SOM = sedimentary organic matter.

Spatial fluctuations of isotopic signatures were heterogeneous among primary producers. δ^13^C signatures were generally significantly higher on intermediate reefs than on other reefs (*H*. *borneensis*, *H*. *cylindracea*, *H*. *discoïdea*, *Turbinaria conoïdes*, *Cymodocea serrulata*). This general pattern also applied to the Grand Sud gradient; no clear pattern was found on the Grand Nouméa gradient (S3 Table). δ^15^N signatures were often significantly lower on intermediate reefs than on other reefs (algal turf, *Halimeda borneensis*, *H*. *discoïdea*, *H*. *opuntia*, *Turbinaria ornata*) (Tables 3 and 4). Once again, this general pattern also applied to some extent to the Grand Sud gradient, whereas on the Grand Nouméa gradient δ^15^N signatures were significantly lower on barrier reefs (algal turf, *H*. *cylindracea*, *H*. *opuntia*, *Halodule uninervis*) (S3 Table). δ^13^C and δ^15^N signatures on intermediate reefs were significantly different from one coast-to-ocean gradient to the other (S3 Table): on the Grand Sud gradient, *H*. *cylindracea* δ^13^C signatures were lower whereas *H*. *borneensis* and *H*. *discoïdea* δ^13^C signatures were higher, and algal turf *H*. *borneensis*, *H*. *cylindracea* and *H*. *opuntia* δ^15^N signatures were lower.

### Seasonal fluctuations of δ^13^C and δ^15^N isotopic signatures

The isotopic signatures of OM pools were relatively stable over time (Table 4). POM remained similar among the reef types across seasons, whereas SOM δ^13^C signatures on barrier reefs were significantly lower in summer (-15.83 ± 2.49 ‰) than in winter (-12.86 ± 2.23 ‰). δ^13^C signatures significantly varied spatially and across seasons, being systematically lower in summer than in winter on Grand Nouméa gradient for fringing reefs, and all reef types of the Grand Sud gradient (S4 Table). Primary producers also displayed a few significant seasonal variations (16 cases among 98 tests; S4 Table). In most cases, isotopic signatures were lower in summer than in winter, apart from the δ^13^C signatures of algal turfs on fringing and intermediate reefs on the Grand Nouméa gradient and the intermediate reef on the Grand Sud gradient.

## Discussion

### Diversity of organic matter sources on coral reefs

The isotopic signatures of OM sources obtained in this study generally fit within typical ranges [34] despite some marginal values (Table 5). For example, the maximal values of δ^13^C signatures for benthic macrophytes and seagrass were above documented ranges, and the minimal values for mangrove δ^15^N signatures were below documented ranges. This concurs with the previously established high variability of benthic primary producers’ δ^13^C signature [15, 27], and significantly help discriminate the OM sources from δ^13^C signatures.

**Table 5:** Ranges of isotopic signatures (δ^13^C and δ^15^N) of major primary producers on aquatic ecosystems following Ostrom and Fry (1993) (a), and comparison with the present study results (b).

Primary producer	δ^13^C (‰)		δ^15^N (‰)	
Marine phytoplankton	-24 to -18 (a)	-30.8 to -16.8 (b)	-2 to 12 (a)	-0.4 to 7.9 (b)
Estuarine phytoplankton	-30 to -15 (a)		2 to 19 (a)	
Benthic macrophytes	-27 to -10 (a)	-22.9 to -2.1 (b)	-1 to 10 (a)	-2.5 to 10.1 (b)
Seagrass	-16 to -4 (a)	-13.2 to -3.5 (b)	0 to 6 (a)	-2.2 to 5.5 (b)
Mangrove trees	-29 to -25 (a)	-31.0 to -25.9 (b)	6 to 7 (a)	3.4 to 7.1 (b)

Despite the high variability of isotopic signatures observed within the main groups, both at the genus and species levels, our results clearly confirm that δ^13^C signature is a efficient and reliable tool to discriminate OM sources in a highly diversified and complex coral reef lagoon as shown in less diversified ecosystems [8, 35]. A wide range of various isotopic signatures among benthic primary producers allows discrimination of the different contributory sources in trophic networks and hence track OM flows. For instance, δ^13^C signatures of coral reef primary producers (algal turf and macroalgae) were clearly distinct from those of associated ecosystems (mangrove and seagrass). Similarly, δ^13^C signatures of the main OM sources were clearly distinct from one another: namely terrestrial (river POM), coastal mangrove and marine (coral reef SOM, algal turf and macroalgae) (Fig 2).

### Origin of OM pools’ isotopic composition

Isotopic signatures may provide useful insights on the relative importance of the various sources on a OM pool’s signature [36]. The Bayesian model showed that POM and SOM pools were influenced by various inputs, different in nature and / or amplitude, and that their relative contributions varied along the coast-to-ocean gradient.

**POM, a pool largely under marine influence.** Links between river inputs and coastal marine POM have been shown on large rivers with strong mean annual flows such as the Rhône river in the Mediterranean [13, 37], and in small rivers influencing fringing reefs in French Polynesia [9]. To our knowledge this paper is the first for coral reef ecosystem.

The influence of river inputs on POM depends on the site location along the coast-to-ocean gradient (Fig 3). Coastal sites are under greater river influence than other sites with a contribution of 50% to reef POM compared to 10% otherwise. Conversely, marine POM contributed to 90%- 95% to POM at other sites. We cannot exclude the possibility that other compounds may influence POM in coastal sites. For instance, degradation of algal and seagrass fragments emerging during low tides or washed onto beaches close to fringing reefs (these cases do not occur on intermediate and barrier reefs) might produce particular compounds that we were not able to take into account. The differences observed between the two gradients could be related to differences in river flows. The Grand Nouméa gradient is under the influence of a river with a mean flow of 11.5 m^3^.s^-1^, against a 3.2 m^3^.s^-1^ mean flow for the river influencing the Grand Sud gradient [38]. The estimated 80 000 m^3^.s^-1^ of marine water entering the SW lagoon of New Caledonia [39] is likely to explain why marine POM was such a strong contributor to coral reef POM. The results did not show any clear influence of *Trichodesmium* spp. blooms on coral reef POM. However, this kind of OM source is difficult to assess as blooms are usually localized in time and space. However, the intensity of the blooms means that at finer temporal and spatial scales, blooms may generate a significant contribution to POM isotopic signatures.

#### SOM, a pool from multiple origins

Influences on SOM also varied along the coast-to-ocean gradient, with a higher contribution of river and mangrove inputs at coastal sites and a higher contribution of seagrass, macroalgae and even *Trichodesmium* spp. at barrier reefs sites (Fig 4). The relative contribution of seagrass to SOM has been demonstrated in other ecosystems [8, 16], and is attributed to compounds of high molecular weight and low degradation rates which are usually trapped in sediments for long periods [40]. Coral reef POM is also known as a significant contributor to SOM, through sedimentation of dead phytoplankton and particulate matter [8, 9]. In the SW lagoon of New Caledonia, the residence time of marine waters ranges from 1 day (barrier reefs) to 3 months (coastal sites) [39]. This may explain the relative importance of marine POM in SOM isotopic composition.

On coastal systems under strong riverine influence the dominant contribution of terrestrial inputs to SOM generates a marked decrease in SOM δ^13^C signature value, which may be statistically undistinguishable to those of river POM [41]. In this study, POM and SOM isotopic compositions were distinct along each site of the coast-to-ocean gradient (Tables 2 and 3). This highlights the limited contribution of riverine POM to SOM, apart from its 15%- 20% contribution to mangrove SOM and coastal SOM. Mangrove leaves clearly contributed to coastal SOM, with up to a 10%- 15% contribution to fringing reefs SOM (compared to negligible contributions for intermediate and barrier reefs). This result highlights transfers of mangrove leaf-derived OM through various coastal ecosystems. Other OM sources, particularly the ones from benthic origins (seagrass, algal turf, and macroalgae) contributed to SOM isotopic signatures, probably through incorporation of their detritus or particular compounds. This contribution was particularly apparent on intermediate and barrier reefs.

### Origin of macrophytes’ isotopic signatures

The ranges of isotopic values of POM and SOM pools and algal turf were relatively narrow compared to the other primary producers (Table 1). Macroalgae and seagrass usually display a high interspecific variability in δ^13^C and a low δ^15^N [8, 42]. This variability in δ^13^C and/or δ^15^N signatures can be high between species belonging to the same genus (*Halimeda* spp.) or remain low (*Turbinaria* spp.), and most likely reflected the macrophyte interspecific functional diversity (*sensu* metabolic / physiological). It remains challenging to better understand the factors driving these contrasting trophic roles.

The isotopic signatures of macrophytes are directly related to their phylogeny, to the biochemical process(es) involved for dissolved inorganic carbon (DIC) uptake during photosynthesis and to environmental characteristics [43]. Phylogeny is generally the main source of variability in δ^13^C signatures among primary producers [44, 45]. However in this study, coral reef macrophytes were hardly distinguishable; Phaeophycea, Rhodophytea and seagrass could not be discriminated by their δ^13^C signatures. Conversely, Chlorophytea appeared distinguishable possibly because *Halimeda* spp. were all calcareous. Only distinct groups (Chlorophytea and Phaeophycea) could be discriminated based on their δ^15^N signatures. This highlights the necessity to investigate phylogeny further to understand what differentiates genera from species. Extra complexity is added when concomitantly examining the photosynthetic processes involved in each species and their sensitivity to environmental conditions.

### δ^13^C, a fingerprint under multiple influences

Macrophytes use different metabolic pathways for DIC uptake in photosynthesis at family, genus, and species levels. Although general trends exist, those of marine primary producers are still subject to clarification and discussion [45]. Two groups are usually identified among Chlorophytea: i) species associated with active CO_2_ uptake and sometimes to Carbon Concentrating Mechanisms “CCM” (δ^13^C signatures comprised between -21 ‰ and -8 ‰), and ii) species associated with CO_2_ diffusion (δ^13^C signatures comprised between -32 ‰ and -25 ‰ [45–47]. Identifying species using HCO_3_^-^ or CO_2_ within Phaeophycea remains challenging as their δ^13^C signatures do not vary much. Rhodophytea δ^13^C signatures are reported to be more heterogeneous [48]; yet caution is necessary, as the origins of the carbon used by species with intermediate δ^13^C signatures remain dubious. One exception is seagrass: their δ^13^C signatures are higher than those of macroalgae highlighting the involvement of a C3 photosynthetic process or possibly the preferential use of HCO_3_^-^ [45].

The range of δ^13^C signatures obtained in this study indicates that the macrophytes (including seagrass) may base their metabolism on an active DIC uptake *via* the use of CCM during photosynthesis [43]. As carbonic anhydrase activity is negatively correlated to δ^13^C values, the more dependent a species is on this metabolic pathway, the higher its δ^13^C signatures [49]. The groups’ averaged δ^13^C signatures suggest that seagrass use these active mechanisms more than Rhodophytea, which use them more than Phaeophycea, which in turn use them more than Chlorophytea.

δ^13^C signatures are usually higher for Chlorophytea, intermediate for Phaeophycea and lower for Rhodophytea [49, 50]. The opposite pattern was obtained in our study and can be explained by several hypotheses: i) the samples composition of genera is likely a key driving-factor of the δ^13^C signatures trends for the main groups of macrophytes. A low group diversity of genera as obtained here, particularly for Chlorophytae with only one genus (*Halimeda)* [49, 50], is known to impact the signature pattern (*e*.*g*. *Halimeda vs*. *Codium* or *Ulva* and *Acanthophora* or *Liagora vs*. *Laurencia* or *Hypnea*; [48–50]); ii) the tropical location of our study site can also be proposed as an explanatory factor. Most macroalgae and seagrass of New Caledonia displayed higher mean δ^13^C signatures than other regions (S5 Table). The CO_2_ diffusion process is negatively correlated with temperature, and surface seawaters in temperate regions generally have higher CO_2_ concentrations and lower δ^13^C values than in seawater from tropical regions [51]. Thus, a photosynthetic organism using DIC *via* a metabolism based on CO_2_ diffusion process will have a lower δ^13^C value in temperate regions than in tropical ones [44, 45, 50]; iii) habitat type and light intensity likely influence the δ^13^C signatures of macrophytes. Environmental parameters such as light intensity are known to influence DIC uptake during photosynthesis and thus impact δ^13^C values [43, 50]. The species with the most ^13^C-depleted signatures (*Halimeda opuntia* and *H*. *heteromorpha*) were mostly encountered on hard substrates, in very shallow waters that are subject to high light intensities. Conversely the species with the most ^13^C-enriched signatures (*Padina australis*, *Liagora* spp., *Turbinaria* spp. and seagrass) were mostly encountered on sandy/detrital substrates, in deeper waters subject to lower light intensities.

### δ^15^N, a fingerprint mostly under local environmental influences

The main taxa were hardly discriminable from their δ^15^N signatures. Chlorophytae still displayed a slightly lower mean δ^15^N signature (1.19 ± 1.34 ‰) than segrass (1.78 ± 1.34 ‰), Rhodophytea (2.51 ± 0.39 ‰), and Phaeophycea (2.93 ± 1.40 ‰). This supports the hypothesis that phylogeny is not a key factor to explain differences in nitrogen uptake processes [52]. In addition, due to a relatively poor knowledge of the enrichment factors associated with inorganic nitrogen uptake it remains more challenging to relate δ^15^N signature to nitrogen inputs in a given site than δ^13^C signatures to DIC [50]. The δ^15^N values of primary producers seem to be related to local environmental characteristics, such as depth [44, 45], anthropogenic activities [53] and river inputs [9].

### Spatial fluctuations of OM sources

Variations in light intensity, temperature or nutriment concentrations may change productivity rates of primary producers and, therefore, their δ^13^C signatures. Similarly, fluctuations in ammonium and nitrate concentrations in seawater can modify δ^15^N signatures of OM sources [15]. These different parameters can vary strongly in coastal shallow ecosystems, potentially causing variability in OM sources δ^13^C and δ^15^N signatures [54].

Our results highlighted a strong spatial variability in the isotopic signatures of OM sources between sites (Table 4). POM isotopic signatures usually closely reflect spatial variations of phytoplankton [55]. This was most likely the case in our study, as POM was strongly influenced by marine inputs and generally coastal / river inputs had a limited influence, except in some coastal sites. The influence of site is more difficult to interpret for primary producers due to their high inter-specific variability in isotopic signatures. The spatial variability of primary producers signatures depends on both the organisms present and the environmental characteristics, such as hydrodynamic conditions or nutrient availability [54, 56].

The signatures of POM and SOM pools, and most macrophytes, were generally ^13^C-depleted and ^15^N-enriched on barrier reefs compared to the other sites. The coast-to-ocean differences in nitrogen-based nutrients concentrations drive patterns of planktonic biomass and generate important modifications in planktonic community structures [57]. This possibly explains the POM variations obtained in our study, where phytoplankton biomasses varied little along the coast-to-ocean gradient [58]. Yet the isotopic patterns remain unexplained. Conversely, clear composition differences across the gradient possibly explain the isotopic patterns observed: micro phytoplankton dominating coastal sites and picoplankton dominating the rest of the lagoon [57]. In addition, POM δ^13^C signatures at coastal and river sites suggest an impact of terrestrial runoff [9]. Nutrient inputs from terrestrial origin only significantly influenced coastal sites [58]. In turn, modification of phytoplanktonic community structure influenced the nutrient cycle [59] and the structure of other food web compartments, such as SOM and benthic primary producers [60]. POM, SOM and primary producers consequently display similar patterns of spatial variability.

Spatial variability observed between the Grand Nouméa and Grand Sud gradients highlighted local characteristics. δ^13^C signatures of fringing reefs POM were higher for Grand Nouméa than Grand Sud. This difference is possibly related to the anthropogenic activities in that zone, such as wastes from a hotel complex and industrial activities in the neighboring bay. SOM and primary producers’ signatures followed a typical pattern along the Grand Sud, but not the Grand Nouméa. On both gradients, SOM and primary producers δ^13^C signatures were low and δ^15^N signatures were high on coastal sites, suggesting an influence of anthropogenic activities and/or terrestrial inputs. Unusually high δ^15^N and low δ^13^C signatures on the Grand Sud barrier reef remain difficult to explain. Despite the absence of data clearly supporting this hypothesis, we suggest that the pattern results from particular hydrologic and/or sedimentary conditions. Examination of spatial variability at such a small scale showed the role of local characteristics, but also demonstrated the necessity to be cautious when attempting to extrapolate local results of δ^13^C and δ^15^N signatures to a wider scale and when interpreting the role of site-dependent environmental factors to assess the transfer of OM from sources to higher trophic levels [56].

### Temporal variations of OM sources

Primary producers’ isotopic signatures are known to fluctuate over time in various ecosystems [12]. Temperature, light intensity, water chemical characteristics, or river runoffs are among the environmental factors that can be involved in such fluctuations [15, 61]. However in our study, temporal variability remained remarkably low over seasons, and mainly concerned δ^13^C signatures with a general trend of lower values during winter. Biogeochemical models indicate that seasonal variations of phytoplankton abundance in the SW lagoon of New Caledonia are mainly explained by nutrients inputs related to rainfall events in the wet season (summer) [58]. In addition, modifications of the phytoplankton community occurred between summer and winter. This implied a change in trophic conditions from mesotrophic to oligotrophic waters [57]. Sporadic events such as hurricanes impacted the amount of nutrients inputs to the lagoon, mostly in coastal zones, but did not alter their isotopic signatures (non-significant differences). Overall, the low freshwater flows and the strong hydrodynamic conditions in the lagoon rapidly homogenized the water composition [58]. SOM and primary producers displayed lower seasonal variations than POM, although δ^13^C and/or δ^15^N seasonal fluctuations have been reported for benthic algae and seagrass [12, 54]. At the seasonal scale of our study, this suggests that variations in temperature or light intensity have a much lower influence on the δ^13^C and/or δ^15^N signatures of primary producers than the homogenization of environmental parameters by hydrodynamic conditions.

### Integration of OM within trophic networks

Most of the studies conducted to assess the relative importance of algae and vascular plants through isotopic analyses highlight algae as the main source of OM for consumers [14, 62]. Their low nutritive value and high lignocellulose concentration make seagrass a second-choice source of food for many herbivorous animals [63]. A high C/N ratio is usually considered as a good proxy for low nutritive value. With a mean C/N ratio of 17.5 (result not shown) seagrass likely constituted an indirect source of OM in coral reef lagoon networks, through detrital processes, accumulation or decomposition within sediments [64, 65]. Conversely, macroalgae and particularly algal turf represent important sources of carbon for some fish [9, 66]. Finally, coral reef POM constitutes a source of OM for planktonic invertebrates and planktonophagous fish.

## Supporting Information

S1 Fig Relative importance of river POM, marine POM and *Trichodesmium* spp. (Tricho.) in the isotopic composition of coral reef POM on fringing, intermediate, and barrier reefs, along the two coast-to-ocean gradients: “Grand Nouméa” and “Grand Sud”. Shaded boxes represent, from dark to light grey, 50%, 75%, and 95% Bayesian credibility intervals.
(TIF)

S1 Table Spatial variations of mean (± sd) isotopic signatures (δ^13^C and δ^15^N) of POM along the two coast-to-ocean gradients ([1] Grand Nouméa: « GN » and [2] Grand Sud: « GS »). Numbers of samples (N) and significance of differences (p) between sites are given. FR = fringing reefs; IR = intermediate reefs; BR = barrier reefs; ns = p> 0.05; * p<0.05; ** p<0.01; *** p< 0.001.
(DOCX)

S2 Table Spatial variations in mean (± sd) isotopic signatures (δ^13^C and δ^15^N) of SOM of the coast-to-ocean gradient on the two zones ([1] Grand Nouméa: « GN » and [2] Grand Sud: « GS »). Numbers of samples (N) and significance of differences (p) between sites are given. FR = fringing reefs; IR = intermediate reefs; BR = barrier reefs; * p<0.05; ** p<0.01; *** p< 0.001.
(DOCX)

S3 Table Summary of spatial variability of primary producers’ isotopic signatures (δ^13^C and δ^15^N) along the general coast-to-ocean gradient and in both zones (Grand Nouméa « GN » and Grand Sud « GS »). Differences between sites and significance (p) are given. FR = fringing reefs; IR = intermediate reefs; BR = barrier reefs; ns = p> 0.05; * p<0.05; ** p<0.01; *** p< 0.001;-: not tested.
(DOCX)

S4 Table Summary of seasonal variability of primary producers’ isotopic signatures (δ^13^C and δ^15^N) along the general coast-to-ocean gradient and on both zones (Grand Nouméa « GN » and Grand Sud « GS »). Differences between sites and significance (p) are given. WI = winter; SU = summer; FR = fringing reefs; IR = intermediate reefs; BR = barrier reefs; ns = p> 0.05; * p<0.05; ** p<0.01; *** p< 0.001;-: not tested.
(DOCX)

S5 Table Mean δ^13^C and δ^15^N signatures (± sd) of some benthic primary producers from various biogeographic regions. See Table 1 for comparison with our results in New Caledonia.
(DOCX)
