# Supplementary figures and images for: Major Sources of Organic Matter in a Complex Coral Reef Lagoon: Identification from Isotopic Signatures (δ13C and δ15N)
Source: PLoS One. 2015 Jul 2;10(7):e0131555. doi: 10.1371/journal.pone.0131555 (PMC4509575; doi:10.1371/journal.pone.0131555)

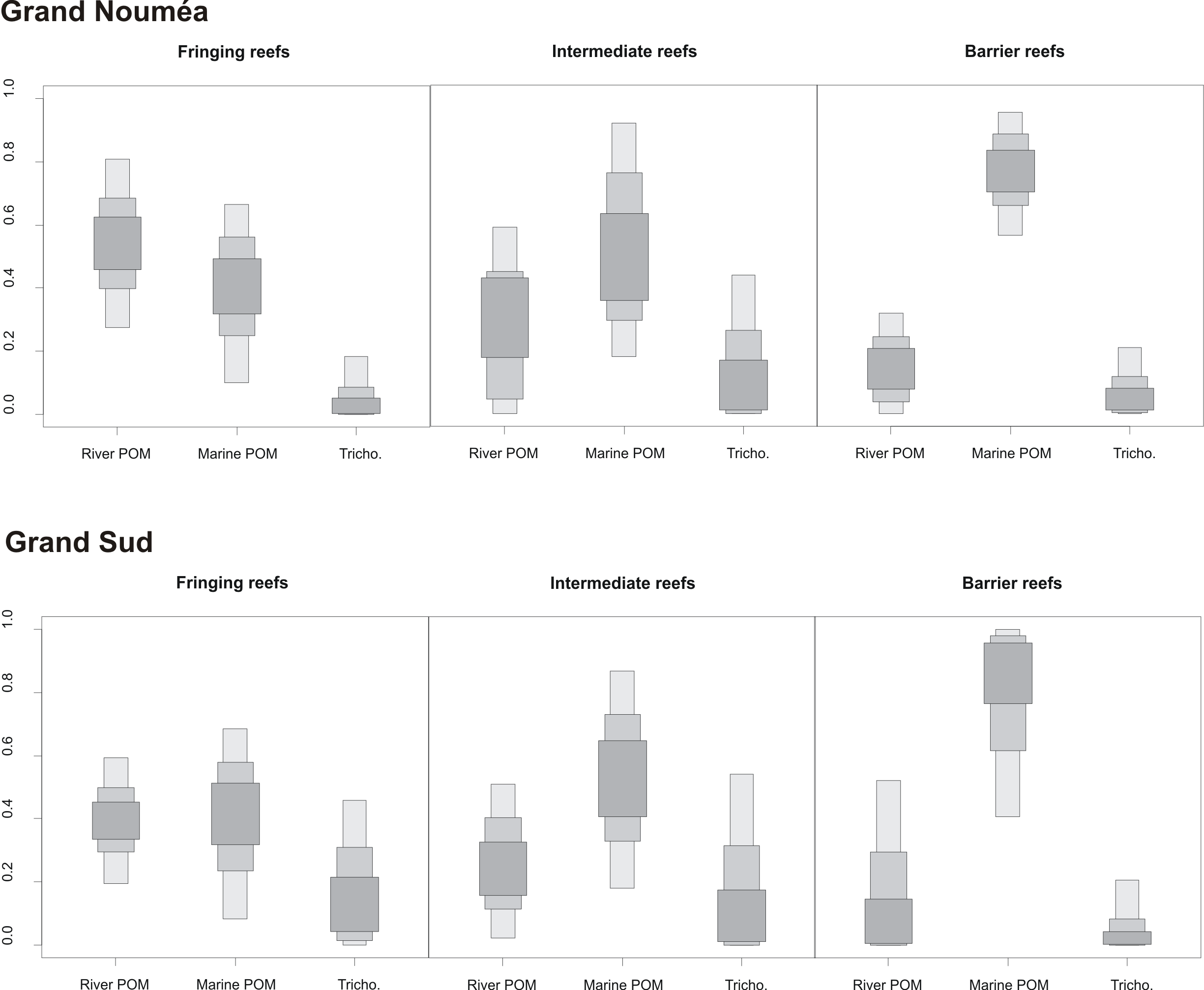

Supplement: S1 Fig — Shaded boxes represent, from dark to light grey, 50%, 75%, and 95% Bayesian credibility intervals. (TIF) [file pone.0131555.s001.tif]
