# Supplementary material for: Major Sources of Organic Matter in a Complex Coral Reef Lagoon: Identification from Isotopic Signatures (δ13C and δ15N)
Source: PLoS One. 2015 Jul 2;10(7):e0131555. doi: 10.1371/journal.pone.0131555 (PMC4509575; doi:10.1371/journal.pone.0131555)
Supplement: S1 Table — Numbers of samples (N) and significance of differences (p) between sites are given. FR = fringing reefs; IR = intermediate reefs; BR = barrier reefs; ns = p> 0.05; * p<0.05; ** p<0.01; *** p< 0.001. (DOCX) [file pone.0131555.s002.docx]

**S1 Table. Spatial variations of mean (± sd) isotopic signatures (δ^13^C and δ^15^N) of POM along the coast-to-ocean gradient on the two zones ([1] Grand Nouméa: « GN » and [2] Grand Sud: « GS »).** Numbers of samples (*N*) and significance of differences (*p*) between sites are given. FR = fringing reefs; IR = intermediate reefs; BR = barrier reefs; ns = p> 0.05; * p<0.05; ** p<0.01; *** p< 0.001.

|  | **Site** | ***N*** | **δ^13^C (**‰) | ***p*** | **δ^15^N (**‰) | ***p*** |
| --- | --- | --- | --- | --- | --- | --- |
| **“GN” gradient** | FR1 | *8* | -17.96 (1.39) |  | 4.74 (0.49) |  |
|  | IR1 | *6* | -19.14 (0.71) | BR1 < FR1 = IR1 *** | 4.07 (0.49) | IR1 < BR1 * |
|  | BR1 | *6* | -21.30 (0.84) |  | 5.20 (0.45) |  |
| **“GS” gradient** | FR2 | *6* | -20.15 (1.22) |  | 4.95 (0.91) |  |
|  | IR2 | *6* | -18.81 (1.81) | BR2 < IR2 * | 3.52 (0.59) | IR2 < FR2 < BR2 * |
|  | BR2 | *6* | -20.34 (2.59) |  | 6.04 (0.78) |  |
