# Supplementary material for: Major Sources of Organic Matter in a Complex Coral Reef Lagoon: Identification from Isotopic Signatures (δ13C and δ15N)
Source: PLoS One. 2015 Jul 2;10(7):e0131555. doi: 10.1371/journal.pone.0131555 (PMC4509575; doi:10.1371/journal.pone.0131555)
Supplement: S2 Table — Numbers of samples (N) and significance of differences (p) between sites are given. FR = fringing reefs; IR = intermediate reefs; BR = barrier reefs; * p<0.05; ** p<0.01; *** p< 0.001. (DOCX) [file pone.0131555.s003.docx]

**S2 Table. Spatial variations in mean (± sd) isotopic signatures (δ^13^C and δ^15^N) of SOM of the coast-to-ocean gradient on the two zones ([1] Grand Nouméa: « GN » and [2] Grand Sud: « GS »).** Numbers of samples (*N*) and significance of differences (*p*) between sites are given. FR = fringing reefs; IR = intermediate reefs; BR = barrier reefs; * p<0.05; ** p<0.01; *** p< 0.001.

|  | **Site** | ***N*** |  | **δ^13^C (**‰) | ***p*** |  | **δ^15^N (**‰) | ***p*** |
| --- | --- | --- | --- | --- | --- | --- | --- | --- |
| **“GN” gradient** | FR1 | *6* |  | -15.76 (0.79) |  |  | 3.75 (0.42) |  |
|  | IR1 | *6* |  | -13.95 (0.48) | FR1 < IR1 < BR1 *** |  | 2.44 (0.27) | IR1 = BR1 < FR1 *** |
|  | BR1 | *7* |  | -12.23 (1.22) |  |  | 2.24 (0.19) |  |
| **“GS” gradient** | FR2 | *7* |  | -14.99 (2.10) |  |  | 3.44 (0.67) |  |
|  | IR2 | *7* |  | -12.79 (0.72) | BR2 < FR2 < IR2 *** |  | 2.16 (0.11) | IR2 < FR2 < BR2 *** |
|  | BR2 | *7* |  | -15.94 (2.79) |  |  | 6.29 (0.66) |  |
