# Supplementary material for: Major Sources of Organic Matter in a Complex Coral Reef Lagoon: Identification from Isotopic Signatures (δ13C and δ15N)
Source: PLoS One. 2015 Jul 2;10(7):e0131555. doi: 10.1371/journal.pone.0131555 (PMC4509575; doi:10.1371/journal.pone.0131555)
Supplement: S3 Table — Differences between sites and significance (p) are given. FR = fringing reefs; IR = intermediate reefs; BR = barrier reefs; ns = p> 0.05; * p<0.05; ** p<0.01; *** p< 0.001;-: not tested. (DOCX) [file pone.0131555.s004.docx]

**S3 Table. Summary of spatial variability of isotopic signatures (δ^13^C and δ^15^N) of primary producers along the general coast-to-ocean gradient and on both zones (Grand Nouméa « GN » and Grand Sud « GS »).** Differences between sites and significance (*p*) are given. FR = fringing reefs; IR = intermediate reefs; BR = barrier reefs; ns = p> 0.05; * p<0.05; ** p<0.01; *** p< 0.001; -: not tested.

| **Primary producers** |  | **General gradient** | **« GN » gradient** | **« GS » gradient** | **GN *versus* GS** |
| --- | --- | --- | --- | --- | --- |
| Algal turf | δ^13^C | ns | ns | ns | ns |
|  | δ^15^N | IR < FR = BR *** | BR1 < FR1 = IR1*** | IR2 < BR2 *** | IR2 < IR1 * |
| *Halimeda borneensis* | δ^13^C | BR < FR = IR *** | IR1 < FR1 ** | BR2 < IR2 *** | IR1 < IR2 *** |
|  | δ^15^N | IR < FR < BR ** | ns | IR2 < BR2 *** | IR2 < IR1 ** |
| *Halimeda cylindracea* | δ^13^C | FR < BR < IR *** | ns | FR2 < BR2 < IR2 ** | IR2 < IR1 *** |
|  | δ^15^N | ns | BR1 < IR1*** | FR2 = IR2 < BR2 ** | IR2 < IR1 ** ; BR1 < BR2 *** |
| *Halimeda discoïdea* | δ^13^C | BR < IR*** | - | BR2 < IR2 ** | IR1 < IR2 *** |
|  | δ^15^N | IR < BR *** | - | IR2 < BR2 * | ns |
| *Halimeda opuntia* | δ^13^C | ns | ns | ns | ns |
|  | δ^15^N | IR < FR < BR ** | BR1 < FR1 = IR1 ** | FR2 = IR2 < BR2 *** | IR2 < IR1** ; BR1 < BR2 *** |
| *Cymodocea serrulata* | δ^13^C | FR < IR ** | FR1 < IR1 ** | - | - |
|  | δ^15^N | ns | ns | - | - |
| *Halodule uninervis* | δ^13^C | ns | ns | - | - |
|  | δ^15^N | BR < IR ** | BR1 < IR1** | - | - |
| *Padina australis* | δ^13^C | ns | - | ns | ns |
|  | δ^15^N | ns | - | ns | ns |
| *Sargassum spinuligerum* | δ^13^C | ns | - | - | - |
|  | δ^15^N | FR < IR ** | - | - | - |
| *Turbinaria conoïdes* | δ^13^C | BR < IR * | BR1 < IR1 * | - | - |
|  | δ^15^N | ns | ns | - | - |
| *Turbinaria ornata* | δ^13^C | ns | - | ns | - |
|  | δ^15^N | IR < BR *** | - | IR2 < BR2 *** | - |
