# Supplementary material for: Major Sources of Organic Matter in a Complex Coral Reef Lagoon: Identification from Isotopic Signatures (δ13C and δ15N)
Source: PLoS One. 2015 Jul 2;10(7):e0131555. doi: 10.1371/journal.pone.0131555 (PMC4509575; doi:10.1371/journal.pone.0131555)
Supplement: S4 Table — Differences between sites and significance (p) are given. WI = winter; SU = summer; FR = fringing reefs; IR = intermediate reefs; BR = barrier reefs; ns = p> 0.05; * p<0.05; ** p<0.01; *** p< 0.001;-: not tested. (DOCX) [file pone.0131555.s005.docx]

**S4 Table. Summary of seasonal variability of isotopic signatures (δ^13^C and δ^15^N) of primary producers along the general coast-to-ocean gradient and on both zones (Grand Nouméa « GN » and Grand Sud « GS »).** Differences between sites and significance (*p*) are given. WI= winter; SU= summer; FR = fringing reefs; IR = intermediate reefs; BR = barrier reefs; ns = p> 0.05; * p<0.05; ** p<0.01; *** p< 0.001; -: not tested.

|  |  | **General gradient** | | | **« GN » gradient** | | | **« GS » gradient** | | |
| --- | --- | --- | --- | --- | --- | --- | --- | --- | --- | --- |
| **Sources** |  | FR | IR | BR | FR1 | IR1 | BR1 | FR2 | IR2 | BR2 |
| POM | δ^13^C | ns | ns | ns | WI < SU ** | ns | ns | WI < SU * | WI < SU ** | WI < SU ** |
|  | δ^15^N | ns | ns | ns | ns | ns | ns | ns | ns | ns |
| SOM | δ^13^C | ns | ns | ns | ns | ns | ns | ns | ns | ns |
|  | δ^15^N | ns | ns | SU < WI *** | ns | ns | ns | ns | ns | ns |
| Turf | δ^13^C | ns | WI < SU *** | SU < WI ** | WI < SU * | WI < SU ** | ns | - | WI < SU ** | SU < WI *** |
|  | δ^15^N | SU < WI * | ns | SU < WI *** | ns | ns | ns | - | ns | SU < WI *** |
| *Halimeda borneensis* | δ^13^C | - | ns | - | - | ns | - | - | - | - |
|  | δ^15^N | - | ns | - | - | ns | - | - | - | - |
| *Halimeda cylindracea* | δ^13^C | SU < WI *** | ns | - | - | - | - | SU < WI *** | ns | - |
|  | δ^15^N | ns | ns | - | - | - | - | ns | ns | - |
| *Halimeda discoïdea* | δ^13^C | - | SU < WI *** | - | - | SU < WI * | - | - | - | - |
|  | δ^15^N | - | SU < WI ** | - | - | SU < WI *** | - | - | - | - |
| *Halimeda opuntia* | δ^13^C | ns | ns | ns | ns | ns | - | - | ns | ns |
|  | δ^15^N | ns | ns | ns | ns | ns | - | - | ns | ns |
| *Halodule uninervis* | δ^13^C | - | ns | ns | - | ns | ns | - | - | - |
|  | δ^15^N | - | SU < WI * | ns | - | SU < WI * | ns | - | - | - |
| *Padina australis* | δ^13^C | - | ns | - | - | - | - | - | ns | - |
|  | δ^15^N | - | ns | - | - | - | - | - | ns | - |
| *Sargassum spinuligerum* | δ^13^C | - | ns | - | - | ns | - | - | - | - |
|  | δ^15^N | - | ns | - | - | ns | - | - | - | - |
