# Supplementary material for: Major Sources of Organic Matter in a Complex Coral Reef Lagoon: Identification from Isotopic Signatures (δ13C and δ15N)
Source: PLoS One. 2015 Jul 2;10(7):e0131555. doi: 10.1371/journal.pone.0131555 (PMC4509575; doi:10.1371/journal.pone.0131555)
Supplement: S5 Table — See Table 1 for comparison with our results in New Caledonia. (DOCX) [file pone.0131555.s006.docx]

**S5 Table. Mean δ^13^C and mean δ^15^N signatures (± sd) of some benthic primary producers from various biogeographic regions.** See Table 1 for comparison with our results in New Caledonia.

| **Species** | **δ^13^C** | **δ^15^N** | **Biogeographical region** | | **Habitat** | **Reference** |  |
| --- | --- | --- | --- | --- | --- | --- | --- |
| **Chlorophytea** |  |  |  |  |  |  |  |
| *Halimeda* spp. | -18.50 (0.50) | 4.50 (0.30) | SW Pacific | New-Caledonia | Coral reefs | Carassou et al. 2008 |  |
| *Halimeda tuna* | -16.3 (0.8) | 5.0 (0.7) | Mediterranean | Italy | Rocky shore | Vizzini et al. 2002 |  |
| **Phaeophycea** |  |  |  |  |  |  | |
| *Cystoseira zosteroides* | -23.58 (1.47) | 3.75 (1.01) | Mediterranean | France | Rocky shore | Cresson 2013 | |
| *Cystoseira balearica* | -16.3 / -13.1 | 1.4 / 3.0 | Mediterranean | Spain |  | Jennings et al. 1997 | |
| *Cystoseira balearica* | -20.35 (0.18) | 2.26 (0.10) | Mediterranean | Corsica | Rocky shore | Pinnegar and Polunin 2000 | |
| *Cystoseira tamarascifolia* | -15.34 (1.12) |  | Mediterranean | Spain | Rocky shore | Mercado et al. 2009 | |
| *Cystoseira barbata* | -16.3 (0.1) | 1.8 (0.2) | Mediterranean | Italy | Rocky shore | Vizzini et al. 2002 | |
| *Cystoseira ercegovicii* | -19.5 (0.1) | 5.4 (0.1) | Mediterranean | Italy | Rocky shore | Vizzini et al. 2002 | |
| *Cystoseira spinosa* | -16.0 (0.2) | 2.2 (0.8) | Mediterranean | Italy | Rocky shore | Vizzini et al. 2002 | |
| *Padina australis* | -7.28 / -6.81 | 3.29 / 5.36 | Central Pacific | Wallis | Coral reefs | Mercader 2013 | |
| *Padina pavonica* | -14.63 (1.43) | 4.76 (0.56) | Mediterranean | France | Rocky shore | Cresson 2013 | |
| *Padina pavonica* | -8.59 (0.18) | 2.91 (0.19) | Mediterranean | Corsica | Rocky shore | Pinnegar and Polunin 2000 | |
| *Padina pavonica* | -13.8 (0.80) | 6.30 (1.20) | Mediterranean | Italy | Rocky shore | Vizzini and Mazzola 2004 | |
| *Padina pavonica* | -11.90 (1.10) | 4.30 (0.80) | Mediterranean | Corsica | Bay | Lepoint et al. 2000 | |
| *Sargassum ilicifolium* | -14.7 / -15.8 |  | NW Pacific | Taiwan | Coral reefs | Wang and Yeh 2003 | |
| *Sargassum vulgare* | -15.40 (0.12) |  | Mediterranean | Spain | Rocky shore | Mercado et al. 2009 | |
| **Rhodophytea** |  |  |  |  |  |  | |
| *Acanthophora spicifera* | -14.6 (0.10) | 0.80 (0.10) | Caribbean | Guadeloupe | Coral reefs | Dromard et al. 2013 | |
| *Acanthophora* sp. | -14.2 (0.01) |  | NW Pacific | Taiwan | Coral reefs | Hsieh et al. 2000 | |
| *Liagora viscida* | -8.53 (1.49) |  | Mediterranean | Spain | Rocky shore | Mercado et al. 2009 | |
| **Seagrass** |  |  |  |  |  |  | |
| *Syringodium isoetifolium* | -5.41 / -4.40 | 1.75 / 3.33 | Central Pacific | Wallis | Coral reefs | Mercader 2013 | |
| *Syringodium filiforme* | -7.1 / -9.4 |  | West Atlantic | Florida |  | Fry 1984 | |
| *Posidonia oceanica* | -12.50 (0.90) | 2.60 (1.00) | Mediterranean | Corsica | Bay | Lepoint et al. 2000 | |
| *Posidonia oceanica* | -14.04 (0.23) | 2.92 (0.04) | Mediterranean | Corsica | Rocky shore | Pinnegar and Polunin 2000 | |
| *Posidonia oceanica* | -12.20 (0.60) | 5.60 (0.60) | Mediterranean | Italy | Rocky shore | Vizzini and Mazzola 2004 | |
| *Posidonia oceanica* | -15.5 / -15.2 | 2.8 / 4.1 | Mediterranean | Italy |  | Vizzini et Mazzola 2006a | |
| *Posidonia oceanica* | -11.3 (0.3) | 2.8 (0.4) | Mediterranean | Italie |  | Vizzini et al. 2002 | |
| *Posidonia oceanica* | -13.2 / -11.4 | 2.8 / 4.1 | Mediterranean | Spain |  | Jennings et al. 1997 | |
| Algal turf | -13.0 / -4.0 | 3.1 (0.20) | SW Pacific | New-Caledonia | Coral reefs | Carassou et al. 2008 | |
| Algal turf | -13.95 / -12.11 | 2.91 / 3.04 | Central Pacific | Wallis | Coral reefs | Mercader 2013 | |
| Algal turf | -18.29 / -13.49 | 3.47 / 5.65 | Central Pacific | Moorea | Coral reefs | Letourneur et al. 2013 | |
| Algal turf | -19.00 / -16.75 | 1.10 / 1.70 | Caribbean | Guadeloupe | Coral reefs | Dromard et al. 2013 | |
